# Perceptions of medical students toward teledermatology as an educational tool: a cross-sectional study

**DOI:** 10.3389/fmed.2023.1206727

**Published:** 2023-06-28

**Authors:** Clara Ureña-Paniego, Alberto Soto-Moreno, Trinidad Montero-Vílchez, Antonio Martínez-López, Agustín Buendía-Eisman, Salvador Arias-Santiago

**Affiliations:** ^1^Department of Dermatology, Hospital Universitario Virgen de las Nieves, Granada, Spain; ^2^Instituto de Investigación Biosanitaria, Granada. IBSGranada, Granada, Spain; ^3^Granada University Medical School, Granada, Spain

**Keywords:** education, teaching, teledermatology, telemedicine, store-and-forward

## Abstract

**Introduction:**

Teledermatology consultations have recently been on the rise, especially due to the SARS-CoV-2 pandemic. The role of teledermatology has been extensively discussed as a mean for the education of dermatology residents. Nevertheless, little has been explored on its use as a pedagogical tool for medical students. The objectives of this study were to assess the level of satisfaction of medical students with teledermatology and to evaluate their opinion about its use as an educational tool.

**Methods:**

A cross-sectional study was carried out at the Dermatology Department, Hospital Universitario Virgen de las Nieves, Granada (Spain). Participants were fourth-year medical students. Every student would spend half of their internship in face-to-face consultations and the other half in teledermatology consultations. Data was collected via self-administered questionnaires.

**Results:**

Eighty one students were finally surveyed, being 66.67% (73/81) female. A majority of students considered a mixed clinical internship model (face-to-face consultations combined with teledermatology) more suitable for obtaining higher marks in the subject of dermatology and in the Medical Intern Resident exam (*p* = 0.04). Nevertheless, face-to-face practice was considered more useful for their training as general practitioners (*p* = 0.04).

**Conclusion:**

Despite the fact that students highly value doctor-patient relationship, teledermatology is considered a powerful educational tool.

## 1. Introduction

Teledermatology consultations have recently been on the rise, especially since the severe acute respiratory syndrome coronavirus 2 (SARS-CoV-2) pandemic (1). Teleconsultations in dermatology allow a greater efficiency and reduction of waiting lists, particularly in a context in which specialists are scarce, healthcare is poorly distributed and population is aging (2–4). Even after the pandemic is slowly fading, the momentum teledermatology gained due to SARS-COV-2 pandemic is likely to linger. The widespread approval of patients and professionals and its acceptable diagnostic accuracy for inflammatory and neoplastic skin conditions contribute to the increasing use of teledermatology (5–8).

Despite all of this, teleconsultations are mostly used with a diagnostic and therapeutic approach, neglecting the educational potential of this tool. Dermatology is an eminently visual field and images from remote consultations could serve this purpose. The power of teledermatology as an educational tool has been tested in residents with promising results (9, 10). Nevertheless, little has been explored on its use as a pedagogical tool for medical students.

Some previous experiences involving medical students in teledermatology practice prove undergraduates value the skills and knowledge telemedicine provides, even more than dermatology residents (10, 11). However, until date, there is no study exploring this topic in our geographical context and in a post-pandemic setting.

In fact, the effect of the SARS-CoV-2 pandemic has heavily affected undergraduate medical education. Internships have been suspended or shifts reduced and pre-clerkship curriculums have transitioned to online formats. Despite the normalization of the pandemic situation, these adaptations have somehow lingered and a major transformation of practical medical education has ensued (12, 13).

The objectives of this study were to assess the level of satisfaction of fourth year medical students with teledermatology and to evaluate their opinion about its use as an educational tool.

## 2. Materials and methods

### 2.1. Study design and participants

A cross-sectional study was conducted. All participants received all available information regarding the conduct of the study and signed a written informed consent form. Eligible population were 4th year medical students of the University of Granada doing an internship at the Dermatology Department, Hospital Universitario Virgen de las Nieves, Granada (Spain) between February 2022 – May 2022. Students who refused to participate in the study were excluded.

### 2.2. Measures

We designed an anonymous, semi-structured self-administered on paper survey in Spanish. Students were assessed about their level of satisfaction of each educational model and its perceived educational value. Students were asked to decide among these models in a number of scenarios. These scenarios were: personal preference, contentedness, perceived learning, suitability for training as general practitioner, for performing better in the subject of Dermatology and for obtaining a higher score in the Medical Intern Resident (MIR) exam. Moreover, surveys included open-ended questions in which students were required to address advantages and disadvantages of the different models in a short open-ended response.

Prior to its distribution, surveys were reviewed by intern and attending physicians for face validity. Responses were manually coded and underwent thematic analysis using the methods described by Braun et al. (14). An English version of the questionnaire is reproduced in S1.

The three educational models were defined as follows: (1) face-to-face model (FTFM): regular dermatology consultations; (2) teledermatology consultations (TDM): store-and-forward system consisting of asynchronous triage, diagnosis and treatment via clinical and/or dermoscopy images sent by primary care physicians to the teledermatology software; and (3) mixed model (MM); the combination of face-to-face and teledermatology consultations. All students underwent the mixed model, spending half of their internship in face-to-face consultations and the other half as active observers of teledermatology consultations. Two subgroups were made. One subgroup underwent face-to-face consultations first and the other initiated the internship with teleconsultations. After completing each modality, students were surveyed.

### 2.3. Statistical analysis

Continuous variables were expressed as means ± standard deviations (SDs) and qualitative variables as absolute and relative frequency distributions. Descriptive statistics were used to evaluate the characteristics of the sample. Normality of the variables was assessed via Shapiro–Wilk test. Continuous variables were expressed as mean and standard deviation (SD). Qualitative variables were presented as relative and absolute frequency distribution. The χ^2^ test was used to compare nominal variables, and the Student’s t-test or was used to compare between continuous data. A *p* value <0.05 was considered statistically significant. Statistical analyses were performed using JMP statistical package version pro 16.0 (SAS Institute, North Carolina, United States).

### 2.4. Ethics statement

Students were warned their participation and answers would not affect their personal evaluations. The study was approved by the ethics committee of Hospital Universitario Virgen de las Nieves.

## 3. Results

One hundred and seventy one students were eventually surveyed but only 81 filled adequately the survey after completing each modality (47.37% response rate). All analyses were conducted on this latter subgroup. 66.67% (54/81) of the responders were female with an average age of 22.4 (2.61) years. Their average academic record was of 7.76 (0.93) in a 0–10 scale grading scale. The average level of satisfaction with this internship was of 8.5 (1.34) in a 0–10 grading scale.

### 3.1. Level of satisfaction and perceived educational value of teledermatology

In our study, 96.79% (78/81) of the participants preferred FTFM internships over TDM (*p* = 0.01) and 56.7% (46/81) considered this model was more useful for their training as general practitioners (*p* = 0.04). Nevertheless, 58.5% (47/81) and 65.41% (53/81) of the students found the MM superior to improve performance in the subject of dermatology (*p* = 0.04) and in the MIR exam (*p* = 0.04), respectively. Differences were statistically significant in all the above comparisons (*p* < 0.05).

**Figure 1:**
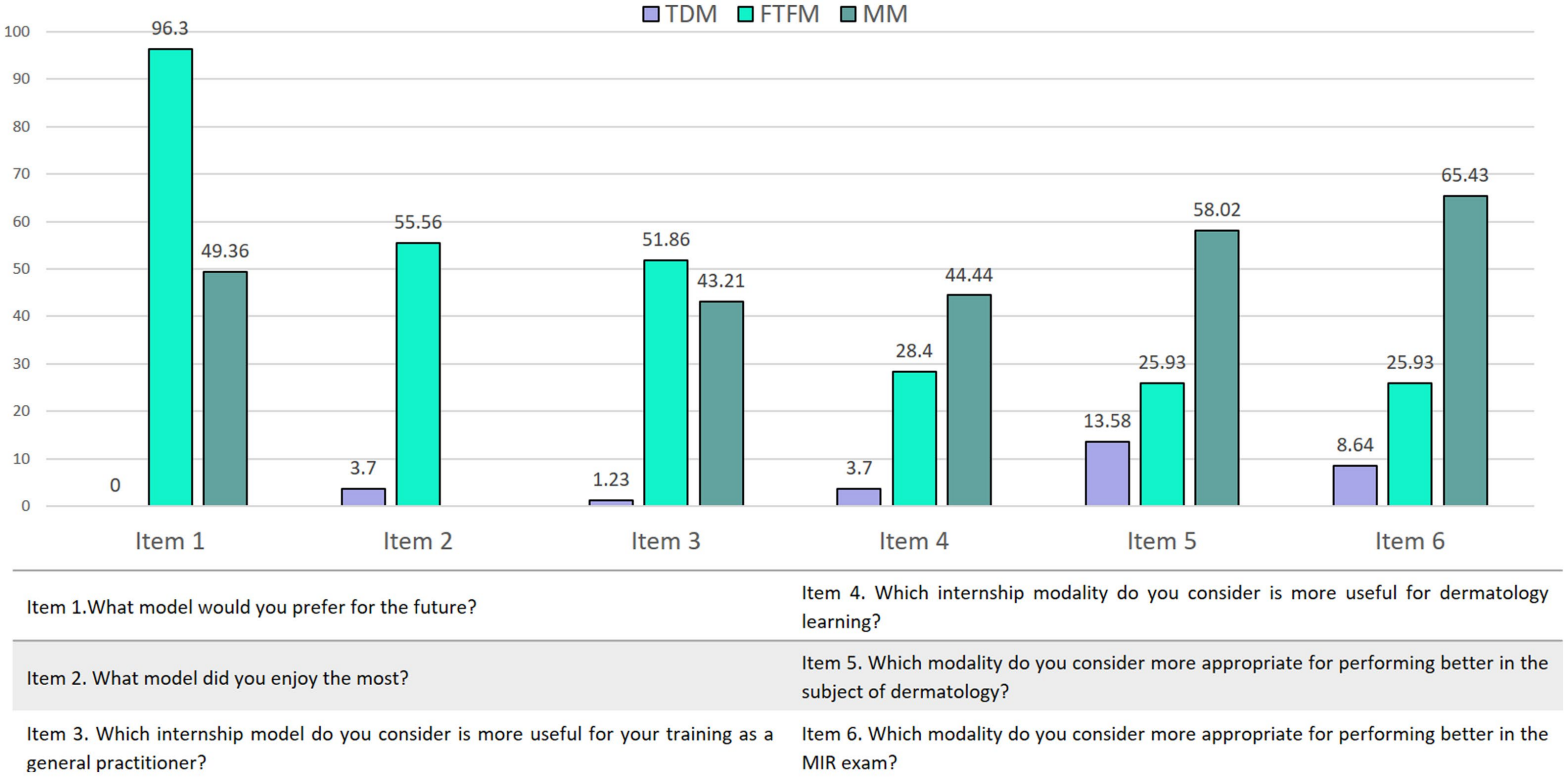
Results of students’ answers across six items: personal preference, contentedness, perceived learning, suitability for training as general practitioner, increasing performance in the subject of dermatology, and in the Medical Intern Resident exam.

Regarding personal preference, 50.63% (41/81) of the students favored the FTFM, without finding significant differences with the MM (*p* = 0.31). Similar results were showed in relation to perceived learning, in which 52.2% (42/81) of the students believed face-to-face consultations were more useful for this purpose but without reaching statistical significance (*p* = 0.49). No differences were either found between the two temporal sequences of the internships (TDM first vs. FTFM first). Additionally, 88.05% (71/81) of participants replied teledermatology was an advantage rather than a barrier in their training. On the other side, 93.04% (75/81) of the students believed building a doctor-patient relationship was more important for their training over learning from a wide variety of clinical cases (Table 1; Figure 1).

**Table 1:** Results of students’ answers across six items: personal preference, contentedness, perceived learning, suitability for training as general practitioner, increasing performance in the subject of dermatology and in the Medical Intern Resident exam.

	TDM	FTFM	MM	*p* value
Item 1.What model would you prefer for the future?	0% (0/81)	50.63% (41/81)	49.37% (40/81)	*P* = 0.31
Item 2. What model did you enjoy the most?	3.7% (3/81)	96.3% (78/81)		*P* = 0.01*
Item 3. Which internship model do you consider is more useful for your training as a general practitioner?	1.23% (1/81)	55.56% (45/81)	43.21% (35/ 81)	*P* = 0.04*
Item 4. Which internship modality do you consider is more useful for dermatology learning?	3.70% (3/81)	51.86% (42/81)	44.44% (36/81)	*P* = 0.49
Item 5. Which modality do you consider more appropriate for performing better in the subject of dermatology?	13.58% (11/81)	28.4% (23/81)	58.02% (47/81)	*P* = 0.04*
Item 6. Which modality do you consider more appropriate for performing better in the MIR exam?	8.64% (7/81)	25.93% (21/81)	65.43% (53/81)	*P* = 0.04*

MIR, Medical Intern Resident; TDM, teledermatology model; FTFM, face-to-face model; MM, mixed model. The asterisk denotes a statistically significant difference between the FTFM and the MM.

### 3.2. Thematic analysis of open-ended questions

For the FTFM, 284 answers were collected: 225 for describing the strengths and 69 for the disadvantages. In this model, 38.6% (83/225) of the answers considered the doctor-patient relationship to be the most valuable asset of the FTFM. This issue was followed by the possibility of performing a complete dermatological examination and the opportunity for students to acquaint themselves with the workflow of a Dermatology department with 20.93% (45/225) and 17.67% (38/225) of the answers, respectively. Students considered insufficient variety of chief complaints (30.43%; 21/69), insufficient internship time (24.64%; 17/69) and a high patient load (15.94%; 11/69) were the major limitations of the FTFM.

On the other side, for TDM, 575 themes were identified: 279 for the strengths and 296 for the barriers of this model. The most frequently mentioned benefits of TDM were the exposure to a wider variety of cases (32.97%; 21/279), knowledge consolidation (27.96%; 78/279) and adjustment to the learning pace of students (17.2%; 48/279). Barriers of the TDM recognized by students were the loss of the doctor-patient relationship (37.2; 110/296), the impossibility of performing a dermatological examination (20.61; 61/296) and the lack of participatory learning strategies (14.2%; 42/296). A more detailed exposition of students’ responses is described in Tables 2, 3.

**Table 2:** Thematic analysis of the advantages and disadvantages of the face-to-face model (FTFM).

FTFM			
Advantages		Disadvantages	
Doctor-patient relationship	36.89% (83/225)	Limited variety of cases	30.43% (21/69)
Performing dermatological examination	20% (45/225)	Insufficient days of internships	24,64% (17/69)
Missing the workflow of a dermatology department	16.89% (38/225)	Low patient load	15.94% (11/69)
Clinical reasoning	16.44% (37/225)	Lack of student involvement	14.49% (10/69)
Student as an active subject	5.78% (13/225)	Theory-practice split	4.35% (3/69)
Discussing clinical questions	2.67% (6/225)	Others	10.14% (7/69)
Incidental diagnosis	0.88% (2/225)		
Effective consultation	0.44% (1/225)		
Total	225	Total	69

**Table 3:** Thematic analysis of the advantages and disadvantages of the teledermatology model (TDM).

TDM			
Advantages		Disadvantages	
Wide variety of cases	32.97% (92/279)	Loss of doctor-patient relationship	37.2% (110/296)
Consolidation of theoretical knowledge	27.96% (78/279)	Impossibility of performing dermatological examination	20.61% (61/296)
Adaptive learning	17.2% (48/279)	Lack of partipatory learning strategies	14.2% (42/296)
Description of skin findings	9.32% (26/279)	Poor image quality	13.51% (40/296)
Convenient for patients	8.96% (25/279)	Excessively theoretical	4.73% (14/296)
Prioritization	6.81% (19/279)	Missing the workflow of a dermatology department	2.36% (7/296)
Collective learning	5.72% (16/279)	Little time dedicated to each individual case	2.02% (6/296)
Dermatological description	9.32% (26/279)	Dehumanization	1.67% (5/296)
Discussing cases without disturbing the patient	3.23% (9/279)	Dependent on technology	1.35% (4/296)
Better performance on MIR exam	2. 51% (7/279)	Less educational	1.35% (4/296)
Interdisciplinary work	1.08% (3/279)	Management of uncertainty	1.01% (3/296)
Good quality images	0.72% (2/279)		
Dermatoscopy learning	0.35% (1/279)		
Total	279	Total	296

There are some excerpts in the surveys of special interest, particularly in relation to teledermatology. Students recognize the potential of teledermatology and its strengths as an educational tool, underlining the importance of telemedicine “*Teledermatology prepares us for a future in which telemedicine will be more prevalent*”; “*It enables filtering skin diseases that do not need to attend in person which is more convenient for doctors and patients*.” Besides, students believe teledermatology practice allows them to be inquisitive and ask questions without disturbing the patient: “*With teledermatology we have the possibility to ask questions without making the patient feel awkward*.” Conversely, participants also identify the perils of teledermatology: “*It distorts doctor-patient relationship and patients become pictures instead of people*”; “*It dehumanizes dermatology*.”

## 4. Discussion

In recent years teledermatology has been the focus of research and clinical practice. Previous studies have proven this tool to be reliable for screening, diagnosis and treatment of skin disorders (4, 5). Besides, it has gained general acceptance among physicians and patients (15). Nevertheless, the opinion of medical students on teledermatology as well as its pedagogical role in their medical training has been less explored. This study highlights the potential of teledermatology as an educational tool and evaluates the level of satisfaction with this model.

The satisfaction among students was high and they reported teledermatology to be useful for their educational program. Remarkably, medical students considered TDM superior to FTFM in more academic setting while valueing the role of FTFM for the development of soft skills. Additionally, no differences were found in perceived learning. This study differs from previous studies in that it focuses on medical students rather than in dermatology trainees and explores their opinions through qualitative analysis.

Boyers et al. (10) reported that dermatology trainees considered TDM to be of great aid regarding increasing their medical knowledge and practice-based learning even despite them considering the doctor-patient relationship to be lacking. Nevertheless, other study conducted in an international setting suggested the opposite, with respondents finding the teledermatology curriculum to be of use for developing their interpersonal and communication skills as physicians, especially regarding cultural and ethical issues in global dermatology as well as the importance of social determinants of health (16).

With most specialties incorporating telemedicine workflows, the inclusion of telemedicine training such as teledermatology for medical students will play an important role in their future career development. Our proposal leverages on existing technological and human infrastructures in the dermatology department. Besides, it does not add to the overflowing curricula of medical school, merging telemedicine and dermatology competencies in an organic fashion.

In our experience, teledermatology proves itself to be valuable for students, especially in the academic context of examinations. Due to its visual nature, students usually get tested on dermatology via a short case with a clinical image. This format resembles that of store-and-forward teledermatology, where a short description is followed by photographs of the patient.

The real strength of teledermatology is its complementary nature to face-to-face consultation. The advantages and disadvantages of both models complement each other when analyzing the student’s replies. This means that a model that combines both could potentiate the strengths of the blended model while minimizing its weaknesses.

It is of interest to point out the importance of humanization in healthcare for new generations of medical students. Other studies show that students are more familiar with telemedicine than employees and value its role in healthcare provision (17, 18). Nevertheless, our results show there is concern regarding dehumanization with this practice and the importance they place on the doctor-patient relationship for becoming a competent doctor. We hypothesize this focus derives from a strong feeling of empathy among our population. Even despite the fact that several authors point to a decline in empathy as medical students transition into their clinical years, the opposite is observed for Spanish medical students (19–21). These sensibilities and the perils of telemedicine and teledermatology should thus, be taken into account.

Almost half of teleconsultations can be managed in a primary care setting, without the need of in-person assessment by a dermatologist (22). This is due to a lower complexity of the complaints referred via teledermatology, in which the most common diagnoses are, according to different authors, seborrheic keratosis, benign nevi and actinic keratosis (22–24). The aforementioned can increase efficiency and prevent patients from traveling long distances for non-threatening skin conditions. Nevertheless, this also implies that when using teledermatology with educational purposes, students will not fully appreciate and grasp the diverse range of dermatological conditions and differential diagnoses. Unless provided with complementary training, students may get a biased impression of dermatology consultations.

Among the limitations of this study is the sample size, which although small, is sufficient to provide some insight on the feelings and emotions medical students might have toward teledermatology. Future directions could explore the role of teledermatology in providing students with practical and valuable knowledge in dermatology, measuring outcomes through quizzing, for example.

Teledermatology has the potential to serve as an educational tool for dermatology teaching and to equip medical students with a comprehensive understanding of the role telemedicine plays in the healthcare system.

## Data availability statement

The raw data supporting the conclusions of this article will be made available by the authors, without undue reservation.

## Ethics statement

The studies involving human participants were reviewed and approved by the Ethics Committee of Hospital Universitario Virgen de las Nieves. The participants provided their written informed consent to participate in this study.

## Author contributions

TM-V, AB-E, and SA-S: concept and methodology. CU-P: software, formal analysis, writing—original draft preparation, and visualization. TM-V and SA-S: validation. CU-P and SA-S: investigation. SA-S: resources and project administration. AM-L and CU-P: data curation. TM-V, AM-L, and SA-S: writing—review and editing. All authors have read and agreed to the published version of the manuscript.

## Conflict of interest

The authors declare that the research was conducted in the absence of any commercial or financial relationships that could be construed as a potential conflict of interest.

## Publisher’s note

All claims expressed in this article are solely those of the authors and do not necessarily represent those of their affiliated organizations, or those of the publisher, the editors and the reviewers. Any product that may be evaluated in this article, or claim that may be made by its manufacturer, is not guaranteed or endorsed by the publisher.
